# The Armadillo as a Model for Leprosy Nerve Function Impairment: Preventative and Therapeutic Interventions

**DOI:** 10.3389/fmed.2022.879097

**Published:** 2022-06-23

**Authors:** Maria Teresa Pena, Ramanuj Lahiri, Gigi J. Ebenezer, Stephen W. Wheat, John Figarola, Richard W. Truman, Linda B. Adams

**Affiliations:** ^1^United States Department of Health and Human Services, Health Resources and Services Administration, Health Systems Bureau, National Hansen’s Disease Program, Baton Rouge, LA, United States; ^2^Department of Neurology, John Hopkins University, Baltimore, MD, United States; ^3^Department of Neurology-Guest Lecturer, Baylor College of Medicine, Houston, TX, United States

**Keywords:** *Dasypus novemcinctus*, *Mycobacterium leprae*, armadillos, leprosy, neuropathies, nerve function impairment

## Abstract

*Mycobacterium leprae* infection of peripheral nerves and the subsequent nerve function impairment (NFI), especially in response to reactional episodes, are hallmarks of leprosy. Improved treatments for *M. leprae*-induced nerve injury are needed, as most if not all of the disability and stigma associated with leprosy arises from the direct or indirect effects of NFI. Nine-banded armadillos (*Dasypus novemcinctus*), like humans, exhibit the full clinical spectrum of leprosy and extensive involvement of the peripheral nerves. In this study, state-of-the-art technology was used to compare nerve function between uninfected and *M. leprae*-infected armadillos. Motor nerve conduction velocity (MNCV) and compound muscle action potential (cMAP), which measure changes in the rate of impulse conduction velocity and amplitude, revealed a progression of impairment that was directly correlated with the duration of *M. leprae* infection and enabled development of an objective nerve impairment scoring system. Ultrasonography accompanied by color Doppler imaging detected enlargement of the *M. leprae*-infected nerves and increased vascularity, possibly due to inflammation. Assessment of epidermal nerve fiber density (ENFD), which shows a length-dependent innervation in armadillos that is similar to humans, identified small fiber degeneration early after *M. leprae* infection. Staining for neuromuscular junction (NMJ) integrity, which is an indicator of signal transduction efficiency into skeletal muscle, discerned a markedly lower number and structural integrity of NMJ in *M. leprae*-infected armadillo footpads. These tools for assessing nerve injury were used to monitor the effects of intervention therapy. Two potential neuro-protective drugs, ethoxyquin (EQ) and 4-aminopyridine (4-AP), were tested for their ability to ameliorate peripheral nerve injury in *M. leprae*-infected armadillos. 4-AP treatment improved MNCV, cMAP, and EFND compared to untreated animals, while EQ had less effect. These results support the armadillo as a model for *M. leprae*-induced peripheral nerve injury that can provide insights toward the understanding of NFI progression and contribute to the preclinical investigation of the safety and efficacy of neuro-preventive and neuro-therapeutic interventions for leprosy.

## Introduction

*Mycobacterium leprae* and the closely related *Mycobacterium lepromatosis* infect the Schwann cells of the peripheral nerves. This infection can result in neuropathy that affects at least a third (1, 2) of the individuals diagnosed with leprosy. Since treatment of the infection often does not lead to resolution of the neuropathy, the number of affected patients is cumulative. Consequently, ∼2 million people currently suffer from leprosy-related nerve function impairment (NFI) (3).

Leprosy neuritis has been well described clinically and histologically for more than 100 years (4–6). In humans, the ulnar, radial, median, and peroneal nerves are primarily involved (7). Robinson et al. (8) reported that the lateral plantar branch of the tibial nerve is also significantly affected. Unfortunately, the mechanisms of nerve injury following *M. leprae* infection are difficult to study in humans as these nerves cannot be biopsied. Therefore, most investigations have been in human, mouse, and rat Schwann cell tissue culture models (9–11) and to a limited extent *in vivo* in mice (12). The mouse, however, may not be a suitable model to study *M. leprae*-induced neuropathy as it is neither naturally susceptible to *M. leprae* infection, nor does it develop any appreciable nerve involvement (7).

Nine-banded armadillos (*Dasypus novemcinctus*), as well as red squirrels (13) and chimpanzees (14), can become naturally infected with *M. leprae* (15–18). Armadillos develop an extensive nerve involvement that closely resembles the neuropathy seen in humans (19, 20). In response to *M. leprae* infection, the majority (>70%) of armadillos show lepromatous-type disease, but borderline and tuberculoid responses are also found (21, 22).

Standardized tests that measure nerve damage are well established for humans (23) and there has been some use of these tests to evaluate neuropathy in persons affected by leprosy (2, 24–28). Validation of such tests in *M. leprae*-infected armadillos would support its use as an animal model to study leprosy NFI. Therefore, we present here several methods to assess peripheral neuropathy in armadillos at early, mid and late stages of leprosy disease. These include motor nerve conduction tests (MNCT), such as motor nerve conduction velocity (MNCV) and compound motor action potential (cMAP), peripheral nerve ultrasonography with Doppler imaging, quantification of epidermal nerve fiber density (ENFD), neuropathic changes in muscle physiological cross sectional area (PCSA), and evaluation of neuromuscular junction (NMJ) number and integrity. Following extensive validation of these tools in both uninfected and *M. leprae*-infected armadillos, two potential neuroprotective drugs were tested in a pilot study to determine the suitability of these methods to evaluate therapeutic interventions to arrest or prevent leprosy neuropathy.

## Materials and Methods

### Armadillos

Wild captured and captive born (genetically identical) nine-banded armadillos were used in this study. The female armadillo gives birth to four genetically identical siblings, which are hand raised and brought to the NHDP after they are weaned. The use of genetically identical siblings enables a higher statistical power for experimental interventions. All animals were conditioned to our facility for a period of 3–6 months (wild captured) or 12 months (captive born). During conditioning, the armadillos were housed in modified rabbit cages and received the following treatments: Penicillin (2 mL IM repeated at 5 days), Ivermectin (0.1 mL SC), Droncit (0.4 mL IM) and Meticorten (0.25 mL IM). At the end of the conditioning period, they were tested for the presence of anti-Phenolic glycolipid-1 (PGL-1) antibodies to determine the presence of pre-existing *M. leprae* infection. Armadillos with pre-existing infection were not used in this study. To determine their immune responsiveness to *M. leprae*, a Lepromin test (heat-killed nude mice footpad derived *M. leprae* strain Thai-53 at 1 × 10^8^ bacilli in 0.1 mL total volume) was administered by intradermal injection to the abdominal skin. After 21 days, the skin test sites were biopsied using a 4 mm biopsy punch and examined histopathologically to score the reactivity of each animal on the Ridley-Jopling scale (29, 30). Only those animals classified as lepromatous (LL) were included in this study.

### Infection of Armadillos With *Mycobacterium leprae*

Armadillos were experimentally infected via intravenous (IV) inoculation of a suspension of 1 × 10^9^ viable *M. leprae* freshly harvested from nude mice footpads (31).

### Anti-pGL-1 Antibody ELISA

PGL-1 antigen (BEI Resources) was used in immunoassays to detect anti-PGL-1 IgM circulating antibody levels in serum samples as previously described (32). Levels of OD > 0.700 (540 nm) were considered positive.

### Light Microscopic and Electron Microscopic Studies

At the time of sacrifice the posterior tibial nerve was collected, fixed in 10% buffered formalin and processed for light microscopic studies, or fixed in 5% glutaraldehyde and processed for electron microscopic studies as previously described (33, 34).

### Motor Nerve Conduction Test

Following anesthesia with 0.4 ml Detomidine and 0.6 ml Ketamine, the animals were placed in a wooden trough ventral side up inside a Faraday cage. Skin temperatures were taken on the medial aspect of each hind limb between the knee and hip to ensure that the MNCT tests were done at body temperatures of 31 ± 2.0°C, because high body temperatures (>35°C) can affect the speed of conduction velocity. An infrared thermometer was used to take the body temperature. We adapted a Cadwell Sierra Summit (Cadwell) portable electrophysiology unit for use with the armadillos. 6.5mm gold cup percutaneous electrodes were prepped with Ten20 conductive paste and used as active, reference, and ground electrodes. After the hind limbs were cleaned with alcohol, the reference electrode was placed at the sulcus of the third toe and the active electrode was placed on an intrinsic muscle between the third and fourth metatarsal bones midshaft. The ground electrode was placed on the anterior/lateral aspect of the hind limb. A cortical disposable stimulator (Natus Neurology) was used to produce a motor stimulus. This stimulator has two 2.2 mm gold plated tips with 5 mm spacing. The negative pole of the stimulator was placed posterior to the medial ankle maleolus for the distal stimulation site. Percutaneous stimulation of the posterior tibial nerve was done near the ankle (distal), and near the knee (proximal) on the medial/posterior shaft of the tibia. Measurements were taken from the proximal stimulation site to the active electrode and from the distal stimulation site to the active electrode to measure the distance between the stimulation sites. Recordings were always done at a supramaximal stimulus. Motor nerve conduction velocity (MNCV) was calculated using the formula: MNCV (in m/s) = (Distance between Proximal and Distal stimulation sites)/(Proximal Latency – Distal Latency). Compound Motor Action Potential (cMAP) amplitude was measured from baseline to negative peak and was recorded in millivolts (mV). Room temperature was maintained between 24 and 26°C throughout the procedure.

### Ultrasonography of the Peripheral Nerves

Posterior tibial nerves were examined via ultrasonography using a SmartUS transducer probe with a linear broadband frequency of 7.5–15 MHz connected to a Sierra Summit basic system (Cadwell). Color Doppler imaging was used to detect blood flow in the posterior tibial nerves. The colors show the speed and direction of blood flow through the vessel. Flow that travels away from the transducer (negative Doppler shift) is depicted in blue. Flow traveling toward the transducer (positive Doppler shift) is depicted in red. Lighter shades of each color denote higher velocities.

### Neuropathic Changes in Muscle Physiological Cross Sectional Area

Following euthanasia, each hind limb was disarticulated at the hip joint and frozen until dissection. The dissection technique used was adapted from Brand et al. (35). The skin, superficial fascia, and fat were removed from the limbs and upper leg muscles were removed for better visualization. Blunt dissection was used to isolate the foot muscles from surrounding tissue. The tendon of insertion was severed distal to the muscle, clamped with a hemostat, and pulled laterally from the tendon of origin. Both tendons were traced into the muscle belly, lightly teasing away the connective tissue holding the muscle in its fusiform shape (Figures 6A,B). This allowed the fibers to align parallel to each other as they course from the tendon of origin to the tendon of insertion. Muscle fibers were measured with a Fowler precision dial caliper. The muscles were severed at their tendon of origin and weighed on an Ohaus scale. The fiber length (in centimeters) and mass (in grams) for each muscle was entered into the following equation to obtain the muscle PCSA: PCSA = Mass/1.02/Fiber length. The PCSA values for each muscle were analyzed along with the MNCT values recorded for the respective hind limb to determine any association.

### Evaluation of Neuromuscular Junctions in the Footpad Muscles

Three uninfected and five *M. leprae*-infected armadillos were used to evaluate integrity and numbers of NMJ in the intrinsic muscles of the hind footpads. Following euthanasia, intrinsic muscles from both hind footpads were carefully dissected and collected in 4% paraformaldehyde at 4°C. After fixing in paraformaldehyde for 4 h, the muscles were incubated overnight in 30% sucrose solution, embedded in OCT (Thermo Fisher Scientific), and stored at -80°C. Thirty μm longitudinal sections were prepared and stained with FITC-labeled Bungarotoxin (Sigma-Aldrich) and PE-labeled anti-myosin antibody (Sigma-Aldrich). A 30 mm^2^ area through the entire thickness of the section was analyzed for the number and integrity of NMJ. Images were captured on an Olympus BX-70 microscope and analyzed using Image Pro software (Dimension, Cellsen).

### Quantification of Epidermal Nerve Fiber Density in the Armadillo Hind Limb

Skin biopsies were obtained from the armadillo hind limb using a 3 mm biopsy punch. The biopsy was fixed in Zamboni solution (Newcomer Supply) for 24 h, washed with 0.08 M Sorenson’s phosphate buffer, fixed in cryoprotectant (0.2 M Sorenson’s phosphate buffer and Glycerol), and stored at -20°C. The biopsies were sectioned with a sliding microtome into 50 μm thick, frozen, vertical, free-floating sections. Four sections were randomly selected and immunostained with rabbit anti-PGP 9.5, a pan axonal marker (Chemicon, Temecula, CA, United States dilution 1:10,000). ENFD were determined following established counting rules (36–38).

### Evaluation of Effectiveness of Neuroprotective Drugs in Preventing and Improving NFI in *Mycobacterium leprae*-Infected Armadillos

Five sets (3 pairs and 2 triplets) of *M. leprae*-infected siblings were randomly allocated into 3 treatment groups as follows: 4-Aminopyridine (4-AP) (*N* = 4), Ethoxyquin (EQ) (*N* = 3) and untreated control (*N* = 5). 4-AP (0.9 mg/day, Sigma-Aldrich) and EQ (2 mg/day, Sigma-Aldrich) were administered orally with the food on a daily basis. The treatment started when the set of animals showed a decrease in cMAP (about 3–7 months post-inoculation) and ended after 60 days. MNCT was performed before, during, and at the end of treatment. At the end of treatment, a 3 mm punch biopsy was collected from the hind leg and fixed in Zamboni fixative. The biopsies were washed, fixed in cryoprotectant, and stored at -80°C for quantification of ENFD.

### Statistical Analysis

Mann–Whitney Rank Sum test and Spearman’s rank test was used to compare differences and correlation, respectively, between different groups using either GraphPad Instat software, version 3.10 (GraphPad Software, Inc.) or Sigma Plot version 12.0 (Systat Software, Inc.). *p* < 0.05 was considered significant.

## Results

A total of 154 armadillos were used in this study, including 36 uninfected and 118 that were experimentally infected with *M. leprae*. All the animals were classified as LL.

### Localization of *Mycobacterium leprae* in the Posterior Tibial Nerve of Infected Armadillos

**FIGURE 1:**
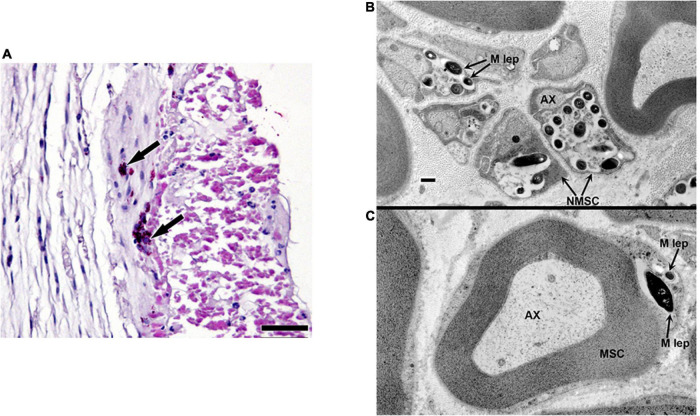
Localization of *M. leprae* in the posterior tibial nerve of infected armadillos. **(A)** Posterior tibial nerve showing clumps of intraneural *M. leprae* (arrows); modified Fite/Faraco stain. Electron micrographs showing *M. leprae* (M. lep) within unmyelinated **(B)** and myelinated **(C)** Schwann cell (SC) located by the axon (AX) in the post-tibial nerve of an armadillo at 24 months post-experimental infection.

Armadillos, like humans, exhibit peripheral nerve involvement upon *M. leprae* infection. Figure 1A depicts a posterior tibial nerve section from a *M. leprae*-infected armadillo showing the presence of intraneural acid-fast bacilli. Transmission electron microscopy shows *M. leprae* located inside the unmyelinated (Figure 1B) and myelinated (Figure 1C) Schwann cells. The effects of this nerve invasion, however, are poorly understood in this model. Therefore, in this study we adapted and applied several non-invasive and invasive tests to evaluate the resultant neuropathy.

### Determination of MNCV and cMAP Values in the Posterior Tibial Nerve of Uninfected Armadillos

**FIGURE 2:**
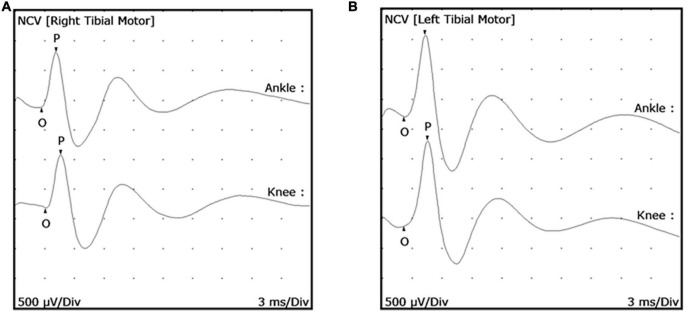
Posterior tibial nerve conduction wave forms in uninfected armadillos. Motor Nerve Conduction Tests (MNCT) were performed on the posterior tibial nerve of uninfected armadillos (*N* = 25) using a Cadwell Sierra Summit portable electrophysiology unit. Representative wave forms are shown of a right posterior tibial **(A)** and left posterior tibial **(B)** nerve. Measurements were taken from the proximal stimulation site to the active electrode and distal stimulation site to the active electrode to measure the distance between stimulation sites. Compound muscle action potential (cMAP) was measured from onset (O) to the peak (P) in both ankle and knee in response to supra-maximal electrical stimulation.

Twenty-five uninfected armadillos were used to develop and standardize MNCT and obtain the normal values for MNCV and cMAP. Upon evaluation of the posterior tibial nerve of each hind limb, a mean MNCV of 62.09 ± 10.72 m/s (range = 88.24–43.75 m/s) was found, with no significant difference between the right and left hind limbs of an individual animal (Figure 2). The mean proximal amplitude was 1.53 ± 0.30 mV (range = 2.12–1.02 mV) and the mean distal amplitude was 1.55 ± 0.33 mV (range = 2.18–0.98 mV). Again, there were no large discrepancies between the right and left cMAP amplitudes of an individual animal. Based on this data we established that a MNCV of <40 m/s (mean - 2 SD) and a cMAP amplitude of <0.9mV (mean - 2 SD) should be considered abnormal for the posterior tibial nerves in armadillos.

### Progression of Abnormal MNCV and cMAP in the Posterior Tibial Nerve of *Mycobacterium leprae*-Infected Armadillos and Development of a MNCT Scoring System

**FIGURE 3:**
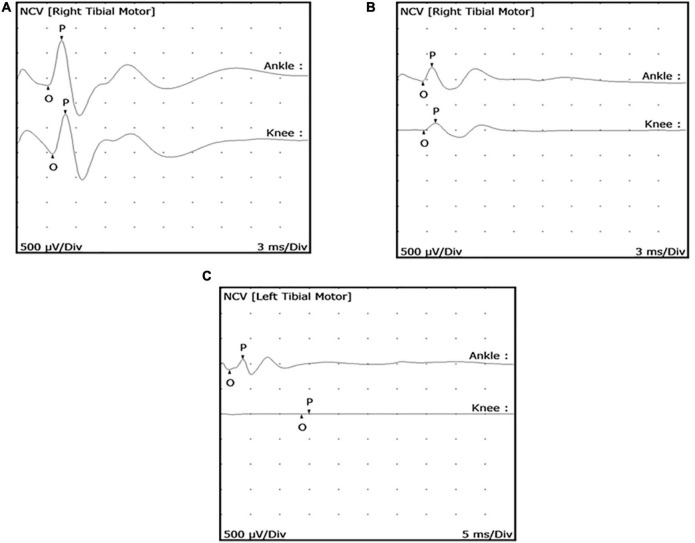
Posterior tibial nerve conduction wave forms showing reduced compound muscle action potential (cMAP) in *M. leprae*-infected armadillos. MNCT were performed on the posterior tibial nerves of *M. leprae*-infected armadillos (*N* = 71). Representative motor nerve conduction wave forms depict early **(A)** (4 months infection) and mid **(B)** (9 months infection) stage leprosy disease and show abnormal wave forms with declining degrees of cMAPs (<0.9 mV). Armadillos progressing to late stage leprosy disease (12–24 months post-inoculation), exhibit conduction block at the knee **(C)**.

*Mycobacterium leprae*-infected armadillos began to exhibit a low cMAP (<0.9 mV) at an early stage of infection (∼4 months post-inoculation) (Figure 3A). As the animals progressed to later stages of leprosy disease they showed a further depressed cMAP (Figure 3B) and eventually complete block of conduction at the knee (Figure 3C). A reduction in cMAP (signifying axonal degeneration) was more common than slowing of MNCV (related to demyelination). Importantly, these results indicated that measurable motor nerve conduction deficit, especially reduced cMAP, could occur early in the course of infection in these animals.

Based on these results we developed the following simple qualitative MNCT scoring system to categorize the armadillos according to their MNCV and cMAP abnormal readings in the posterior tibial nerve. The animals with both normal MNCV and cMAP in both hind limbs were arbitrarily assigned a score of 5. Each abnormality, low MNCV or cMAP in each hind limb, was considered as 1-point, which was substracted from the normal score of 5 to obtain the MNCT for a particular animal as follows:

Score 5: animals having no abnormal MNCV or cMAP in either hind limbs.
Score 4: animals having only one abnormality, either slow MNCV (<40 m/s) or low cMAP (< 0.9mV) in only one hind limb.
Score 3: animals having any combination of two abnormal readings in the MNCV and/or cMAP.
Score 2: animals having any combination of three abnormal readings in the MNCV and/or cMAP.
Score 1: animals having abnormal MNCV and cMAP in both right and left hind limbs.

Therefore, an animal showing both abnormal MNCV and cMAP in both hind limbs will have a score of 5–4 = 1, the lowest possible in this system.

### Correlation of MNCT With Disease Progression and Anti-PGL-1 Antibody Levels

**FIGURE 4:**
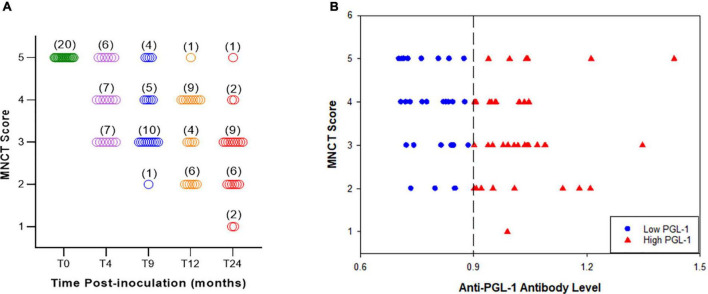
MNCT scores decrease with duration of infection and correlate with anti-PGL1 antibody titers. A longitudinal study **(A)** demonstrated negative correlation between the MNCT score and duration of *M. leprae* infection, indicating progressive nerve injury (*N* = 20). **(B)** Phenolic glycolipid I (PGL-1) antigen was used in an enzyme immunoassay to detect anti-PGL1 IgM circulating antibody levels in armadillo serum samples. Armadillos with high (>0.900 OD 540 nm) anti-PGL1 antibody titers have lower MNCT scores, while those with low (0.700–0.900 OD 540 nm) anti-PGL-1 antibody titers have higher MNCT scores (*N* = 71).

A set of 20 *M. leprae*-infected armadillos were individually evaluated for NFI throughout their leprosy disease progression using the MNCT scoring system standardized above. Figure 4A shows the MNCT scores of individual animals at pre-inoculation and at 4, 9, 12, and 24 months post-inoculation. At time 0 (uninfected) all the animals show a score of 5 with no abnormalities in the MNCV or cMAP in either hind limb. The scores began to decrease as early as 4 months post-infection with the majority of animals showing low scores at the late stages of leprosy disease (12 and 24 months post-infection).

We have previously shown that antibody levels for the *M. leprae*-specific PGL-1 correlate with bacterial load in armadillos (39–41). In order to determine the correlation between anti-PGL-1 antibody levels and impaired nerve conduction, we stratified 71 *M. leprae*-infected armadillos at different stages of leprosy disease into two groups according to serum anti-PGL-1 IgM antibody levels. As shown in Figure 4B, sera with OD 540nm values between 0.700 and 0.900 were considered as a low (+) anti-PGL-1 titer group (30/71) while sera with OD values of >0.900 were considered as a high (++) anti-PGL-1 titer group (41/71). Overall, 80.3% (57/71) of infected armadillos developed measurable motor nerve conduction abnormality in at least one of their posterior tibial nerves (MNCT score < 5). Within the low anti-PGL-1 group, 73.3% (22/30) showed impaired nerve conduction in at least one hind limb while in the high anti-PGL-1 group, 85.4% (35/41) showed impaired nerve conduction (Figure 4B). Although no significant difference (*p* = 0.3297) was found in impaired nerve conduction between low and high PGL-1 groups, conduction abnormality among *M. leprae*-infected armadillos generally tends to increase with rising anti-PGL-1 antibody levels. In the low anti-PGL-1 group, 36.6% (11/30) of the animals had a score of 3 or below while in the high anti-PGL-1 group 61.0% (25/41) of the animals had a score of 3 or below. In addition, nearly all of the animals that developed conduction deficit also eventually exhibited signs of clinical neuropathy in their footpads. Decreased cMAP correlated well with the number of wounds and heavy calluses (r = -0.72, *p* = 0.02) on the plantar surface of the foot (data not shown). The occurrence of hypertrophic nails and nail avulsion also tend to increase along with decreasing cMAP and increased anti-PGL-1 antibodies.

### Ultrasonography of the Posterior Tibial Nerves

**FIGURE 5:**
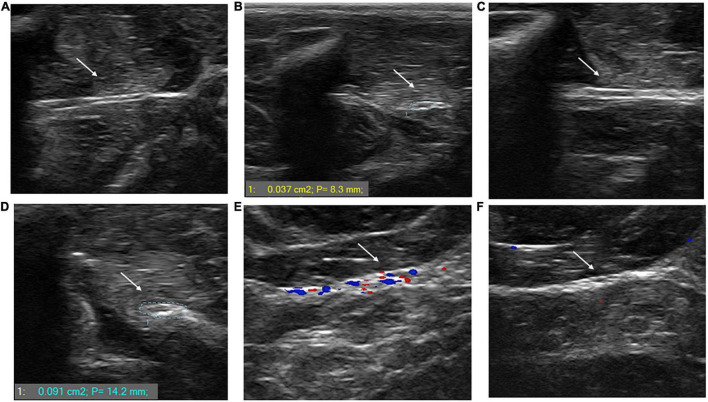
*M. leprae* infection causes nerve enlargement in armadillo peripheral nerves. Ultrasonography of the posterior tibial nerve was performed using a SmartUS transducer probe with a linear broadband frequency of 1.5–15 MHz connected to a Sierra Summit basic system. The posterior tibial nerve is shown along its longitudinal **(A)** and transverse **(B)** axes in an uninfected armadillo and a *M. leprae*-infected armadillo [**(C,D)**, respectively]. The nerve diameter of the *M. leprae*-infected armadillo is 0.096 cm^2^ indicating nerve enlargement compared to the nerve diameter of the uninfected armadillo (0.037 cm^2^). Ultrasound with color Doppler imaging in *M. leprae*-infected armadillos showed increased blood flow in animals at late stage leprosy disease (23 months post-infection) that were positive for anti-PGL-1 antibodies **(E)** compared to armadillos that were negative for anti-PGL-1 antibodies at 9 months post-infection **(F)**. The intense colors indicate lower velocity of the blood flow traveling away and toward the transducer (*N* = 4).

Figure 5 shows ultrasonography of the posterior tibial nerve in transverse and longitudinal axes of uninfected (A and B) and *M. leprae*-infected (C and D) armadillos at an early stage (4 months post-inoculation) of leprosy disease. The posterior tibial nerve of the *M. leprae*-infected armadillo was larger when measured at the transverse axis (0.096 cm^2^; P: 13.1 mm) compared to the uninfected armadillo (0.037 cm^2^; P: 8.3 mm). Nerve enlargement can also been seen when examined at the longitudinal axis. Figure 5E shows the ultrasonography of the posterior tibial nerve of an *M. leprae*-infected armadillo at a late stage of leprosy disease. Increased blood flow, indicating possible inflammation, was seen using color Doppler imaging in an armadillo at 23 months post*-M. leprae* inoculation. Interestingly, this animal had a high level of anti-PGL-1 IgM antibody (2.104 at OD 540 nm). No increased blood flow (5F) was seen in an armadillo at a mid-stage disease (9 months post-inoculation) that was negative for anti-PGL-1 IgM antibody (0.225 at OD 540 nm).

### Neuropathic Changes in Muscle Physiological Cross Sectional Area

**FIGURE 6:**
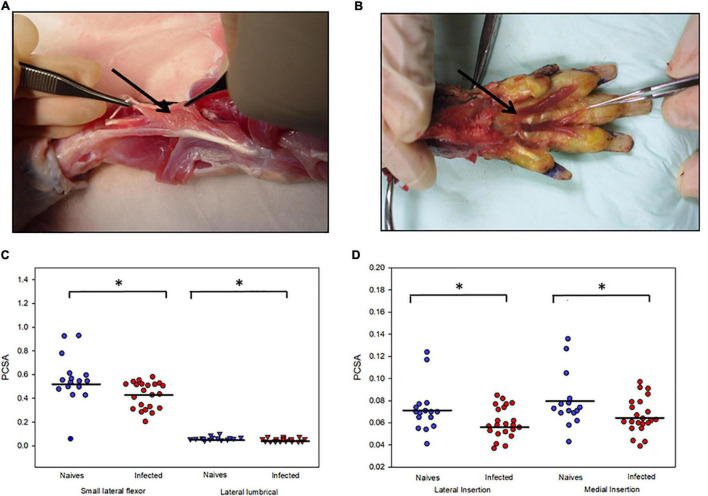
*M. leprae* infection causes atrophy of armadillo footpad muscles. Photographs of the dissection of a representative animal showing **(A)** the small lateral flexor muscle of the footpad and how the fibers run between the tendons and **(B)** the lumbrical muscles of the right hind limb that were released from their tendon of insertion. **(C)** Atrophy of small lateral flexor and lateral lumbrical muscles in *M. leprae*-infected armadillos compared to uninfected armadillos. **(D)** Atrophy of medial and lateral insertions of the medial lumbrical muscle in *M. leprae*-infected armadillos (*N* = 10) compared to uninfected armadillos (*N* = 8). *p < 0.05.

We examined the PCSA of footpad muscles of 18 armadillos, of which 10 were infected with *M. leprae* and 8 were uninfected (Figures 6A,B). The PCSA values of the small lateral flexor, lateral lumbrical (Figure 6C), and both insertions (medial and lateral) of the medial lumbrical muscles (Figure 6D) of the *M. leprae*-infected armadillos averaged 20% lower than that of the uninfected animals (*p* < 0.02). Furthermore, there were statistical differences (*p* = 0.008) between MNCT scores between the groups.

### Integrity of the Neuromuscular Junction in the Footpad Muscles

**FIGURE 7:**
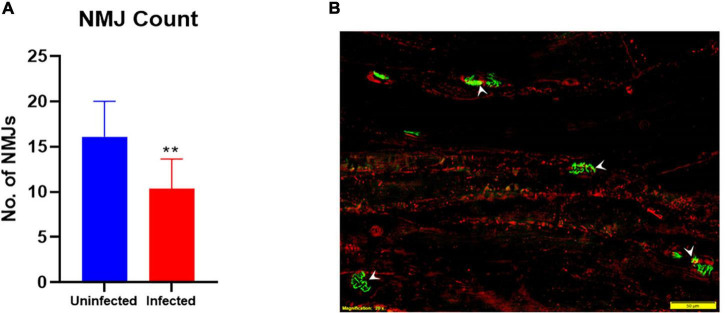
*M. leprae* infection causes degeneration of neuromuscular junctions (NMJ’s) in the armadillo footpad muscles. The number of NMJs **(A)** is reduced (**p = 0.0012) in *M. leprae*-infected armadillos (terminal stage) as measured in 30μm-thick, 30^2^ mm muscle sections. Fluorescent micrograph **(B)** of footpad intrinsic muscle in an *M. leprae*-infected armadillo showing NMJs (green, arrowheads) stained with FITC-α-bungarotoxin and muscle fibers (red) stained with PE-anti-myosin. (*N* = 8).

Intrinsic muscle from the hind footpads of 3 uninfected and 5 *M. leprae*-infected late-stage armadillos were used to quantify the numbers and fragmentation of post-synaptic NMJ. A significant reduction (*p* = 0.0012) in the number of NMJs was observed in this muscle in *M. leprae*-infected armadillos (Figure 7A). Furthermore, ∼50% of the NMJs in the infected armadillos (Figure 7B) were fragmented when compared to those in uninfected animals.

### Evaluation of Effect of Neuroprotective Drugs in Preventing and Improving Nerve Function Impairment in *Mycobacterium leprae*-Infected Armadillos

**FIGURE 8:**
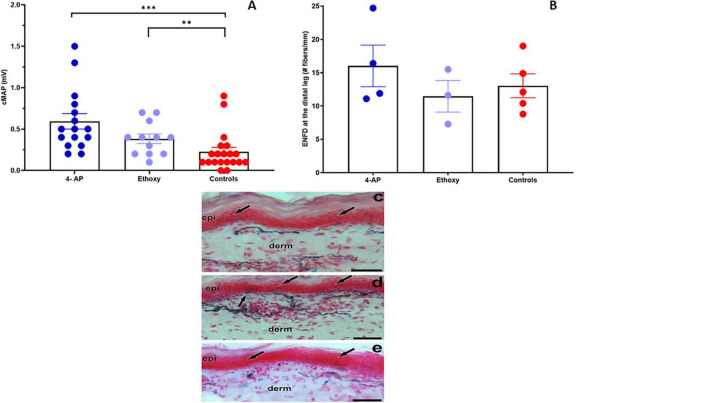
Evaluation of the effect of neuroprotective drugs on NFI in *M. leprae* infected armadillos. **(a)** Armadillos treated with 4-Aminopyridine- (4-AP) (*N* = 4) and Ethoxyquin (EQ) (*N* = 3) showed a significantly higher (***p = 0.001 and **p = 0.012, respectively) cMAP (mV) than the untreated controls (*N* = 5) at 1 month post-treatment. **(b)** Epithelial nerve fiber density (ENFD, #fibers/mm) in 4-AP- and EQ-treated and untreated armadillos. **(c)** Skin section from an untreated armadillo stained with PGP9.5 showing epidermal nerve fibers (arrows) terminating at the epidermis (epi). **(d)** Skin section from a 4-AP-treated armadillo showing more branching of the epidermal nerve fibers (arrows) and that the dermal nerve bundles are dense at the papillary dermis (derm) in comparison to the untreated armadillo. **(e)** Skin section from an EQ treated armadillo showing single epidermal nerve fibers (arrows) entering the epidermis. Scale bars = 50 μm.

Motor nerve conduction studies together with ENFD for sensory nerves were used to evaluate the ability of two neuroprotective drugs, 4-AP and EQ, to improve nerve function impairment in *M. leprae*-infected armadillos. Electrophysiological studies indicated that armadillos treated with 4-AP and EQ showed a significantly higher (*p* = 0.001 and *p* = 0.012, respectively) cMAP than the untreated controls at 1 month post-treatment (Figure 8a). No significant differences were seen in the cMAP between EQ and 4-AP treated animals (*p* = 0.105). At the end of the treatment all the untreated (N = 4) and EQ (N = 3) treated animals had a MNCT score of 3 while the 4-AP treated animals (N = 3) yielded MNCT scores of 3, 4 and 5. Similarly, armadillos treated with 4-AP showed higher ENFD than untreated controls and EQ treated armadillos; however, the differences in this limited number of animals did not reach significance (Figure 8b). Figure 8c depicts a skin section from an *M. leprae*-infected armadillo stained with PGP.9.5, showing fewer branching of the epithelial nerve fibers compared to a 4-AP-treated armadillo (Figure 8d). EQ-treated armadillo also shows fewer branching than 4-AP-treated armadillo (Figure 8e).

## Discussion

The lack of an effective animal model to study *M. leprae*-induced nerve injury has greatly hampered understanding of the mechanisms involved. Nine-banded armadillos are naturally susceptible to *M. leprae* infection (16, 17) and show the full histopathological spectrum of leprosy. More importantly, armadillos develop extensive peripheral nerve involvement due to *M. leprae* infection that closely resembles neuropathy in humans (19, 42). Previous histopathological studies performed in the armadillo posterior tibial nerve suggested that the localization of *M. leprae* in the peripheral nerve proceeds from outside in as opposed to an ascending-type of infection. Similar to human LL cases, infected armadillo nerves demonstrate a heavy infiltration of *M. leprae*-loaded nucleated cells in many fascicles of nerve trunks and invading demyelinating Schwann cells (5, 42, 43). However, due to the exotic nature of this animal model, no standardized methods are available to detect and accurately measure the degree of neuropathy or nerve damage in armadillos. Standardized tools to study the progression of neuropathy as well as to evaluate any therapeutic interventions are essential to advancing the armadillo as a model for NFI. The purpose of this study was to establish multiple non-invasive tests, like MNCT, ENFD and ultrasonography, which could be performed on the same animal to follow disease progression in the peripheral nerves. We also evaluated muscular atrophy and NMJ integrity in the footpad muscles as end-point assessments for the degree of neuro-muscular damage at the terminal stage of the disease. Together these tests form a robust set of tools to evaluate peripheral neuropathy in *M. leprae*-infected armadillos.

Neuropathies in humans are diagnosed using peripheral motor and sensory nerve conduction studies that evaluate the integrity of nerve-muscle and the somatosensory circuit (23). Motor nerve conduction studies are commonly performed by electrically stimulating (percutaneous) a peripheral motor or mixed nerve and recording the electrical response from a suitable muscle innervated by the same nerve. Analysis of the conduction velocity (MNCV), negative peak amplitude (cMAP), and the shape of the wave form following electrical stimulation reveal the degree of demyelination and axonal loss in the studied segment of the peripheral motor or mixed nerve. Demyelination results in slowing of conduction velocity measured in meters/second (m/s). In some cases, demyelination can also cause dispersed cMAP. In contrast, axonal loss leads to a decrease in the cMAP measured in millivolts (mV) (27). Since percutaneous nerve conduction studies are non-invasive, they are convenient ways to study onset and progress of peripheral neuropathy over time in the same subject.

Although the hard carapace and thick skin limit the number of nerves that may be examined in armadillos, we have developed and validated MNCT techniques and an MNCT scoring system that can be used to assess motor nerve conduction in both hind limbs by examining the posterior tibial nerve that lies beneath the skin surface between the ankle and knee and innervates the small lumbrical and flexor muscles of each foot. Similar to that seen in humans, leprosy is asymmetrical and manifests randomly in different nerves in these animals. Moreover, like in humans there is a high probability that the posterior tibial nerve will become involved, and that probability increases as the disease progresses (20, 42). We have shown in previous electron microscopy studies that posterior tibial nerves of armadillos progressing at 28 months post infection exhibited *M. leprae* within both myelinated and unmyelinated axons and Schwann cells. The axons showed edema with demyelination and axonal degenerative changes (20, 43).

Peripheral conduction deficit among *M. leprae*-infected armadillos began early in the course of their disease and progressed over time. Decreased cMAP amplitude (<0.9mV), which indicates axonal loss, was the most common impairment. *M. leprae*-infected armadillos rarely showed dispersed cMAP, which also indicates axon loss, but abnormal nerve conduction velocity (MNCV < 40 m/s) was observed. Some studies have reported demyelination as the main cause of nerve dysfunction in leprosy disease (44–46). Another study, however, found that axonal injuries occurred at a higher frequency than myelin injuries in the posterior tibial nerve (8). Hence, the sequence of events leading to nerve injury may vary among different nerves or an individual’s position in the clinical spectrum. 70% (14/20) of experimentally infected armadillos developed demonstrable conduction deficit in at least one of their posterior tibial nerves within 4 months of infection, and its onset generally coincided with detectable anti-PGL-1 IgM antibodies. Eventually, nearly all *M. leprae*-infected armadillos will exhibit neuropathy as their disease disseminates. As the disease progressed, both right and left posterior tibial nerves were compromised showing delayed MNCV, which is a consequence of demyelination of the nerve fibers, and in some cases total conduction block. Armadillos in very late stages of experimental leprosy may develop lesions in their footpads that can make MNCT difficult to perform or can produce inaccurate results. Extending our electrophysiological techniques to include sensory nerve and F-wave responses will likely increase the sensitivity of detecting peripheral neuropathy and will likely reveal even greater involvement of nerves at these early stages of infection. Nevertheless, our data clearly shows that in armadillos there is a significant association between the onset of the host immune response (anti-PGL-1 antibodies) and conduction abnormalities in the peripheral nerves of the lower extremities which progresses over time to manifest classical lesions in the foot.

Peripheral nerves are often enlarged in leprosy patients and ultrasonography is currently used to determine nerve enlargement (28, 47, 48). It provides an objective measure of the nerve dimensions in addition to revealing structural changes over a longer length of the nerve (49). Enlarged nerves of patients undergoing leprosy reactions show increased endoneurial and epineurial vascularity that can be measured by ultrasonography with color or power Doppler (24, 28). We also used ultrasonography of the posterior tibial nerve to show that armadillos, like humans, show enlargement of peripheral nerves due to *M. leprae* infection. Although leprosy reactions have not yet been determined in armadillos, increased vascularity, most likely due to inflammation, could be demonstrated using color Doppler (49). Unlike MNCT, ultrasonography can be done on multiple peripheral nerves of upper and lower extremities of the animal, thereby increasing the probability of detecting neuropathy in focal and asymmetric disease progression.

Monitoring warm/cold detection or grip dynometry are currently among the most effective early indicators of nerve injury in human leprosy, and these tests often show changes well before conduction abnormality is apparent (2, 35). Unfortunately, armadillos cannot grip objects and they are generally unresponsive to common temperature stimuli. However, Brand showed that the PCSA (cross sectional area/mass) of muscles in the leprotic hand could be used as a surrogate measure of grip strength and index muscle atrophy (35). We examined the PCSA of footpad muscles of *M. leprae*-infected and uninfected armadillos and found that the infected armadillos showed significant muscle atrophy indicated by lower fiber density compared to the uninfected armadillos. Additionally, we compared neuromuscular junction (NMJ) integrity and numbers in footpad intrinsic muscles, between end stage *M. leprae*-infected and uninfected armadillos and found significant loss of NMJs in infected armadillos. Further studies are required to understand the mechanisms of motor NMJ innervations and decay dynamics in *M. leprae*-infected armadillos and to evaluate reconnection of the NMJ’s, which can give important insights into possible therapeutic interventions to arrest or reverse loss of neuro-muscular function in leprosy.

Motor nerve conduction and sensory tests together with ultrasonography are suitable for evaluation of peripheral nerve function. Since these are non-invasive tests they can be used to follow disease progression in experimentally infected armadillos and consequently to evaluate efficacies of therapeutic and immune interventions in preventing or reversing peripheral nerve damage. Within sensory tests, quantification of ENFD is a test that is used to diagnose small fiber neuropathies including leprosy induced neuropathies (50). In small fiber sensory neuropathies associated with diabetes, HIV and idiopathic small fiber neuropathies, a decrease in epidermal density in the distal leg has been demonstrated (51–53). We have previously reported that armadillos show a length dependent innervation similar to humans (42, 50); therefore, this test can be used to diagnose small fiber neuropathies associated with *M. leprae*-infection in armadillos. Using MNCT, it was demonstrated that Lepvax, a vaccine against leprosy, showed some nerve protection in *M. leprae*-infected armadillos, indicated by higher cMAP recordings and restoration of axonal size of the tibial nerve fibers in vaccinated armadillos compared to the unvaccinated (43).

In the current study, we evaluated two neuroprotective drugs that have been used successfully in cancer models to ameliorate neuropathy following chemotherapy (54) and in acute peripheral nerve injury models promoting remyelination (55). EQ, a quinolone-based antioxidant, has been shown to prevent axonal loss in peripheral nerves of mice with chemotherapy-induced neurotoxicity. EQ provided a dose-dependent neuroprotection indicated by higher intraepidermal nerve fiber density and sensory nerve potential amplitude, and lower thermal hypoalgesia compared to the untreated controls (54). A second drug, 4-AP, has been used in Lambert-Eaton myasthenic syndrome and multiple sclerosis and acts by blocking potassium channels, prolonging action potentials, and increasing neurotransmitter release at the neuromuscular junction. It was also shown to be effective in promoting remyelination and axon regeneration in acute peripheral nerve injuries. In clinical trials, administration of 4-AP improved multiple neurological signs of multiple sclerosis such as vision, muscle strength and coordination (56, 57). 4-AP treatment in acute peripheral injury enhanced both the speed and extent of restoration of normal NCV and caused regenerative increases in axonal area, myelin thickness, and levels of the myelin-specific protein (55). Both EQ and 4-AP were effective in preventing demyelination and axon loss, and treatment resulted in higher epidermal nerve fiber density (ENFD) indicating higher sensory integrity in treated animals (54, 55). We used motor nerve conduction studies together with sensory tests (ENFD) to assess the effect of these two neuroprotective drugs (4-AP and EQ) in preventing and or improving nerve function impairment in *M. leprae*-infected armadillos in a limited pilot study. After one month of treatment, 4-AP improved motor nerve function in armadillos that were progressing at an early stage of leprosy disease, indicating that this drug merits further evaluation in this model. This pilot study shows that the armadillo model and the various nerve structure and function assessment methods validated in this study have the required sensitivity to evaluate efficacy of therapeutic interventions against peripheral neuropathy in leprosy.

In summary, multiple non-invasive and invasive methods to evaluate *M. leprae*-induced peripheral NFI in nine-banded armadillos were validated, and a quantitative nerve deficit scoring system was developed. The data clearly indicate that the armadillo lower extremity becomes heavily involved and exhibits many of the same abnormalities as seen in humans. This study further suggests that, like humans, armadillos suffer an insidious silent neuropathy that commences early in the infection, and serological screening can be useful to stage the disease status for nerve injury studies. These techniques detected pathological events at early stages of disease and will benefit future studies to elucidate mechanisms involved in early leprosy neuropathy. Moreover, the armadillo model and these tests to evaluate peripheral neuropathy will support the screening of new preventive interventions for leprosy control such as leprosy specific vaccines and chemotherapies to either prevent, arrest or reverse nerve damage.

## Data Availability Statement

The raw data supporting the conclusions of this article will be made available by the authors, without undue reservation.

## Ethics Statement

The animal study was reviewed and approved by the NHDP Institutional Animal Care and Use Committee (Assurance #D16-000019 [A3031-01]) and was performed in accordance with the United States Department of Agriculture and Plant Health Inspection Service.

## Author Contributions

MP and RL validated MNCT test and ultrasonography of peripheral nerves and analyzed the data. GE counted and analyzed the EFND data. JF was instrumental in validating the MNCT test. SW validated the ultrasonography in peripheral nerves in armadillos. LA and RT conceived the study and secured funding. MP, RL, and LA wrote the manuscript. All authors provided critical reviews and approved the submitted version.

## Author Disclaimer

The views expressed in this article are solely the opinions of the authors and do not necessarily reflect the official policies of the U. S. Department of Health and Human Services or the Health Resources and Services Administration, nor does mention of the department or agency names imply endorsement by the U.S. Government.

## Conflict of Interest

The authors declare that the research was conducted in the absence of any commercial or financial relationships that could be construed as a potential conflict of interest.

## Publisher’s Note

All claims expressed in this article are solely those of the authors and do not necessarily represent those of their affiliated organizations, or those of the publisher, the editors and the reviewers. Any product that may be evaluated in this article, or claim that may be made by its manufacturer, is not guaranteed or endorsed by the publisher.

## References

[1] Rodrigues LC Lockwood D. Leprosy now: epidemiology, progress, challenges, and research gaps. *Lancet Infect Dis.* (2011) 11:464–70. 10.1016/S1473-3099(11)70006-8 21616456

[2] van Brakel WH Nicholls PG Wilder-Smith EP Das L Barkataki P Lockwood DN. Early diagnosis of neuropathy in leprosy-comparing diagnostic tests in a large prospective study (the INFIR Cohort Study). *PLoS Negl Trop Dis.* (2008) 2:e212. 10.1371/journal.pntd.0000212 PMC2270341 18382604

[3] World Health Organization [WHO]. *Weekly Epidemiological Record. World Health Organization Weekly Epidemiological Record.* Vol. 36. Genva: World Health Organization (2021). p. 421–44.

[4] Ebenezer GJ Scollard DM. Treatment and evaluation advances in leprosy neuropathy. *Neurotherapeutics.* (2021) 18:2337–50. 10.1007/s13311-021-01153-z 34799845 PMC8604554

[5] Scollard DM. The biology of nerve injury in leprosy. *Lepr Rev.* (2008) 79:242–53. 19009974

[6] Vijayan J Wilder-Smith E. Chapter 2.5: Neurological manifestations of leprosy. In: Scollard DM Gillis TP editors. *International Textbook of Leprosy.* Greenville, SC: American Leprosy Missions (2016).

[7] Scollard DM Adams LB Gillis TP Krahenbuhl JL Truman RW Williams DL. The continuing challenges of leprosy. *Clin Microbiol Rev.* (2006) 19:338–81. 10.1128/CMR.19.2.338-381.2006 16614253 PMC1471987

[8] Robinson R Alexandre P Kirchner D Garbino H. Nerve Conduction studies of the tibial nerve across the tarsal tunnel in leprosy patients. *Hansenol Int.* (2015) 40:3–8.

[9] Hagge DA Oby Robinson S Scollard D McCormick G Williams DL. A new model for studying the effects of Mycobacterium leprae on Schwann cell and neuron interactions. *J Infect Dis.* (2002) 186:1283–96. 10.1086/344313 12402198

[10] Rambukkana A Zanazzi G Tapinos N Salzer JL. Contact-dependent demyelination by Mycobacterium leprae in the absence of immune cells. *Science.* (2002) 296:927–31. 10.1126/science.1067631 11988579

[11] Tapinos N Rambukkana A. Insights into regulation of human Schwann cell proliferation by Erk1/2 via a MEK-independent and p56Lck-dependent pathway from leprosy bacilli. *Proc Natl Acad Sci U S A.* (2005) 102:9188–93. 10.1073/pnas.0501196102 15967991 PMC1166596

[12] Shetty VP Antia NH. Light and ultrastructural study of sciatic nerve lesions induced using intraneural injection of viable Mycobacterium leprae in normal and immunosuppressed Swiss white mice. *Int J Lepr Other Mycobact Dis.* (2002) 70:25–33. 12120037

[13] Avanzi C Del-Pozo J Benjak A Stevenson K Simpson VR Busso P Red squirrels in the British Isles are infected with leprosy bacilli. *Science.* (2016) 354:744–7. 10.1126/science.aah3783 27846605

[14] Hockings KJ Mubemba B Avanzi C Pleh K Dux A Bersacola E Leprosy in wild chimpanzees. *Nature.* (2021) 598:652–6. 10.1038/s41586-021-03968-4 34646009 PMC8550970

[15] da Silva MB Portela JM Li W Jackson M Gonzalez-Juarrero M Hidalgo AS Evidence of zoonotic leprosy in Para, Brazilian Amazon, and risks associated with human contact or consumption of armadillos. *PLoS Negl Trop Dis.* (2018) 12:e0006532. 10.1371/journal.pntd.0006532 PMC6023134 29953440

[16] Job CK Sanchez RM Hunt R Truman RW Hastings RC. Armadillos (*Dasypus novemcinctus*) as a model to test antileprosy vaccines; a preliminary report. *Int J Lepr Other Mycobact Dis.* (1993) 61:394–7. 8228437

[17] Truman R Sanchez R. Armadillos: models for leprosy. *Lab Anim.* (1993) 22:28–32.

[18] Vera-Cabrera L Ramos-Cavazos CJ Youssef NA Pearce CM Molina-Torres CA Avalos-Ramirez R Mycobacterium leprae Infection in a Wild Nine-Banded Armadillo, Nuevo Leon, Mexico. *Emerg Infect Dis.* (2022) 28:747–9. 10.3201/eid2803.211295 35202538 PMC8888246

[19] Pena M Sharma R Truman R. Chapter 10.2: The armadillo model for leprosy. In: Scollard DM Gillis TD editors. *International Textbook of Leprosy.* Greenville, SC: American Leprosy Missions (2016).

[20] Sharma R Lahiri R Scollard DM Pena M Williams DL Adams LB The armadillo: a model for the neuropathy of leprosy and potentially other neurodegenerative diseases. *Dis Model Mech.* (2013) 6:19–24. 10.1242/dmm.010215 23223615 PMC3529335

[21] Adams LB Pena MT Sharma R Hagge DA Schurr E Truman RW. Insights from animal models on the immunogenetics of leprosy: a review. *Mem Inst Oswaldo Cruz.* (2012) 107(Suppl 1):197–208. 10.1590/s0074-02762012000900028 23283472

[22] Rosa PS Belone AF Silva EA. Mitsuda reaction in armadillos *Dasypus novemcinctus* using human and armadillo derived antigens. *Hansenol Int.* (2005) 30:180–4.

[23] Delisa J Lee H Baran EM. *Manual of Nerve Conduction Velocity and Clinical Neurophysiology in Lippincott.* Philadelphia, PA: Williams and Wilkins (1994).

[24] Wheat SW Stryjewska B Cartwright MS. A hand-held ultrasound device for the assessment of peripheral nerves in leprosy. *J Neuroimaging.* (2020) 31:76–8. 10.1111/jon.12797 33176039

[25] Shrestha BK Ranabhat K Pant R Sapkota S Shrestha S. Neuritic Leprosy; An intriguing re-visit to a forbidden ailment. *Kathmandu Univ Med J (KUMJ).* (2019) 17:73–6. 31734684

[26] Akita J Miller LHG Mello FMC Barreto JA Moreira AL Salgado MH Comparison between nerve conduction study and high-resolution ultrasonography with color doppler in type 1 and type 2 leprosy reactions. *Clin Neurophysiol Pract.* (2021) 6:97–102. 10.1016/j.cnp.2021.02.003 33869903 PMC8047122

[27] Falck B Stalberg E. Motor nerve conduction studies: measurement principles and interpretation of findings. *J Clin Neurophysiol.* (1995) 12:254–79. 11221785

[28] Kumaran M Thapa M Narang T Prakash M Dogra S. Ultrasonography versus clinical examination in detecting leprosy neuropathy. *Leprosy Rev.* (2019) 90:364–70.

[29] Job CK Truman RW. Mitsuda-negative, resistant nine-banded armadillos and enhanced Mitsuda response to live M. leprae. *Int J Lepr Other Mycobact Dis.* (1999) 67:475–7. 10700925

[30] Narayanan RB Girdhar A Girdhar BK Malaviya GN. Immunohistological analysis of nerve granulomas in neuritic leprosy. *Int Arch Allergy Appl Immunol.* (1990) 92:50–5. 10.1159/000235223 2246076

[31] Truman RW Krahenbuhl JL. Viable M. leprae as a research reagent. *Int J Lepr Other Mycobact Dis.* (2001) 69:1–12. 11480310

[32] Duthie MS Truman RW Goto W O’Donnell J Hay MN Spencer JS Insight toward early diagnosis of leprosy through analysis of the developing antibody responses of Mycobacterium leprae-infected armadillos. *Clin Vaccine Immunol.* (2011) 18:254–9. 10.1128/CVI.00420-10 21177914 PMC3067361

[33] Job CK McCormick GT Scollard DM Truman RW. Electron microscope appearance of lepromatous footpads of nude mice. *Int J Lepr Other Mycobact Dis.* (2003) 71:231–9. 10.1489/1544-581x(2003)71<231:emaolf>2.0.co;2 14608819

[34] Wallace E Hendrickson D Tolli N Mehaffy C Pena M Nick JA Culturing Mycobacteria. *Methods Mol Biol.* (2021) 2314:1–58. 34235647 10.1007/978-1-0716-1460-0_1

[35] Brand PW Beach RB Thompson DE. Relative tension and potential excursion of muscles in the forearm and hand. *J Hand Surg Am.* (1981) 6:209–19. 10.1016/s0363-5023(81)80072-x 7240676

[36] Ebenezer GJ Hauer P Gibbons C McArthur JC Polydefkis M. Assessment of epidermal nerve fibers: a new diagnostic and predictive tool for peripheral neuropathies. *J Neuropathol Exp Neurol.* (2007) 66:1059–73. 10.1097/nen.0b013e31815c8989 18090915

[37] Lauria G Hsieh ST Johansson O Kennedy WR Leger JM Mellgren SI European Federation of Neurological Societies/Peripheral Nerve Society Guideline on the use of skin biopsy in the diagnosis of small fiber neuropathy. Report of a joint task force of the European Federation of Neurological Societies and the Peripheral Nerve Society. *Eur J Neurol.* (2010) 903-912:e944–909. 10.1111/j.1468-1331.2010.03023.x 20642627

[38] McArthur JC Stocks EA Hauer P Cornblath DR Griffin JW. Epidermal nerve fiber density: normative reference range and diagnostic efficiency. *Arch Neurol.* (1998) 55:1513–20. 10.1001/archneur.55.12.1513 9865794

[39] Job CK Drain V Williams DL Gillis TP Truman RW Sanchez RM Comparison of polymerase chain reaction technique with other methods for detection of Mycobacterium leprae in tissues of wild nine-banded armadillos. *Lepr Rev.* (1991) 62:362–73. 10.5935/0305-7518.19910042 1784151

[40] Truman RW Morales MJ Shannon EJ Hastings RC Evaluation of monitoring antibodies to PGL-1 in armadillos experimentally infected with *M. leprae. Int J Lepr.* (1986) 54:556–9. 3546545

[41] Zhou Z Pena M van Hooij A Pierneef L de Jong D Stevenson R Detection and monitoring of Mycobacterium leprae infection in nine banded armadillos (*Dasypus novemcinctus*) Using a Quantitative Rapid Test. *Front Microbiol.* (2021) 12:763289. 10.3389/fmicb.2021.763289 PMC8581735 34777319

[42] Truman RW Ebenezer GJ Pena MT Sharma R Balamayooran G Gillingwater TH The armadillo as a model for peripheral neuropathy in leprosy. *Institute Lab Anim Res J.* (2014) 54:304–14. 10.1093/ilar/ilt050 24615444 PMC4158350

[43] Duthie MS Pena MT Ebenezer GJ Gillis TP Sharma R Cunningham K LepVax, a defined subunit vaccine that provides effective pre-exposure and post-exposure prophylaxis of M. leprae infection. *NPJ Vaccines.* (2018) 3:12. 10.1038/s41541-018-0050-z PMC5871809 29619252

[44] Andrade PR Jardim MR da Silva AC Manhaes PS Antunes SL Vital R Inflammatory cytokines are involved in focal demyelination in leprosy neuritis. *J Neuropathol Exp Neurol.* (2016) 75:272–83. 10.1093/jnen/nlv027 26888306

[45] Job CK. Pathology of peripheral nerve lesions in lepromatous leprosy–a light and electron microscopic study. *Int J Lepr Other Mycobact Dis.* (1971) 39:251–68. 5169800

[46] Shetty VP Antia NH Jacobs JM. The pathology of early leprous neuropathy. *J Neurol Sci.* (1988) 88:115–31. 10.1016/0022-510x(88)90210-9 2852213

[47] Lugao HB Frade MA Marques W Jr. Foss NT Nogueira-Barbosa MH. Ultrasonography of leprosy neuropathy: a longitudinal prospective study. *PLoS Negl Trop Dis.* (2016) 10:e0005111. 10.1371/journal.pntd.0005111 PMC5112942 27851766

[48] Nagappa M Visser LH Bathala L. Peripheral nerve sonography, a novel technique fro improving the diagnosis of Hansen’s neuropathy. *Leprosy Rev.* (2021) 92:202–6.

[49] Jain S Visser LH Praveen TL Rao PN Surekha T Ellanti R High-resolution sonography: a new technique to detect nerve damage in leprosy. *PLoS Negl Trop Dis.* (2009) 3:e498. 10.1371/journal.pntd.0000498 PMC2716078 19668356

[50] Ebenezer GJ Pena MT Daniel AS Truman RW Adams L Duthie MS Mycobacterium leprae induces Schwann cell proliferation and migration in a denervated milieu following intracutaneous excision axotomy in nine-banded armadillos. *Exp Neurol.* (2022) 352:114053. 10.1016/j.expneurol.2022.114053 35341747 PMC9019856

[51] Holland NR Stocks A Hauer P Cornblath DR Griffin JW McArthur JC. Intraepidermal nerve fiber density in patients with painful sensory neuropathy. *Neurology.* (1997) 48:708–11. 10.1212/wnl.48.3.708 9065552

[52] Periquet MI Novak V Collins MP Nagaraja HN Erdem S Nash SM Painful sensory neuropathy: prospective evaluation using skin biopsy. *Neurology.* (1999) 53:1641–7. 10.1212/wnl.53.8.1641 10563606

[53] Polydefkis M Hauer P Sheth S Sirdofsky M Griffin JW McArthur JC. The time course of epidermal nerve fibre regeneration: studies in normal controls and in people with diabetes, with and without neuropathy. *Brain.* (2004) 127:1606–15. 10.1093/brain/awh175 15128618

[54] Zhu J Carozzi VA Reed N Mi R Marmiroli P Cavaletti G Ethoxyquin provides neuroprotection against cisplatin-induced neurotoxicity. *Sci Rep.* (2016) 6:28861. 10.1038/srep28861 27350330 PMC4924091

[55] Tseng KC Li H Clark A Sundem L Zuscik M Noble M 4-Aminopyridine promotes functional recovery and remyelination in acute peripheral nerve injury. *EMBO Mol Med.* (2016) 8:1409–20. 10.15252/emmm.201506035 27861125 PMC5167128

[56] Davis FA Stefoski D Rush J. Orally administered 4-aminopyridine improves clinical signs in multiple sclerosis. *Ann Neurol.* (1990) 27:186–92. 10.1002/ana.410270215 2317014

[57] Krishnan AV Kiernan MC. Sustained-release fampridine and the role of ion channel dysfunction in multiple sclerosis. *Mult Scler.* (2013) 19:385–91. 10.1177/1352458512463769 23077091

